# Tolerance in Thalamic Paraventricular Nucleus Neurons Following Chronic Treatment of Animals with Morphine

**DOI:** 10.1523/ENEURO.0249-24.2025

**Published:** 2025-06-06

**Authors:** Omar Koita, Joseph J. Lebowitz, John T. Williams

**Affiliations:** Vollum Institute, Oregon Health & Science University, Portland, Oregon 97239

**Keywords:** desensitization, morphine, neuronal excitability, opioids, tolerance, withdrawal

## Abstract

Neurons in the paraventricular nucleus of the thalamus (PVT) integrate visceral and limbic inputs and project to multiple brain regions to bias behavior toward aversive or defensive states. This study examines MOR signaling in anterior PVT neurons in brain slices from untreated and morphine-treated animals. Imaging in a MOR-Cre reporter rat revealed extensive expression in aPVT cells, and the application of [Met]^5−^ enkephalin (ME) induced outward currents which were abolished by the MOR-selective antagonist CTAP. A saturating concentration of ME resulted in desensitization that was blocked by compound 101, indicating a phosphorylation-dependent process. The opioid sensitivity of amygdala-, nucleus accumbens-, and prefrontal cortex-projecting neurons was then examined. Neurons that projected to the amygdala were more sensitive to ME than cortical- and accumbal-projecting cells. Following chronic treatment, tolerance to morphine was found in neurons projecting to the amygdala and nucleus accumbens with a trend toward tolerance observed in neurons projecting to the prefrontal cortex. The results reveal that adaptations to chronic opioid exposure are in the aPVT circuits contribute to affective pain processing and may provide specific insights into the etiology of withdrawal following the cessation of opioid use.

## Significance Statement

The PVT connects visceral sensation to cognitive and limbic nuclei with a particularly strong role in pain processing. Nearly all neurons in the anterior PVT (aPVT) neurons express opioid receptors, suggesting that this brain region likely contributes to affective and cognitive facets of opioid analgesia and dependence. Understanding the acute actions and adaptations induced by opioids in the PVT therefore provide insight into affective pain processing and potentially connect it to the affective withdrawal symptoms seen in opioid use disorder.

## Introduction

The analgesic tolerance seen following sustained opioid exposure is mediated by adaptive processes that counter the continued activation of opioid receptors on both rapid and extended timescales. These include receptor-dependent tolerance, marked by a decrease in the coupling of receptors to effectors that reduces signaling efficacy. Phosphorylation of μ-opioid receptors (MORs), which leads to the recruitment of β-arrestin and triggers receptor desensitization and internalization, is critical for receptor-dependent tolerance (Williams et al., 2013). However, receptor-dependent tolerance is cell type specific, with a range of adaptations observed across distinct brain regions (Levitt and Williams, 2018; Birdsong and Williams, 2020). Adaptive processes beyond receptor tolerance include changes in neuronal excitability, synaptic plasticity, and circuit-level adaptions that oppose the continued presence of opioids. Upon termination of opioid exposure, these adaptive processes reverse more slowly than receptor inactivation, often revealing hypertrophic signaling believed to contribute to symptoms of withdrawal. The causal contributions of withdrawal symptoms to opioid use disorder, particularly those that involve affective processing, remain under intense investigation (Koob, 2013; Koob and Volkow, 2016). Thus, understanding the acute and chronic actions of opioids in brain regions that participate in affective pain processing represent a critical locus in the understanding of opioid use disorder and the emergence of withdrawal symptoms.

The paraventricular nucleus of the thalamus (PVT) is a midline thalamic structure that integrates information from subcortical (i.e., limbic) and cortical (i.e., cognitive) nuclei (Li and Kirouac, 2012; Kirouac, 2015; Otis et al., 2019; Penzo and Gao, 2021). The PVT plays a crucial role in emotional and motivational aspects of behavior including arousal, stress, and reward processing, and it has been implicated in the actions of opioids and opioid dependence (Bengoetxea et al., 2020; Chisholm et al., 2020; Keyes et al., 2020; Giannotti et al., 2021; Yu et al., 2021; Babaei et al., 2023; Zhu et al., 2023; McDevitt et al., 2024; Paniccia et al., 2024). PVT signaling contributes to changes in arousal (Gao et al., 2020; Bu et al., 2022; Zhao et al., 2022; Eacret et al., 2023; Duan et al., 2024) and pain awareness (Chang et al., 2019; Zhang et al., 2022, 2023) associated with opioid withdrawal, signifying specific contributions to the widely distributed phenomenon. Previous studies have shown that both spontaneous and antagonist-induced withdrawal increase c-*fos* expression in PVT neurons (Chahl et al., 1996; Zhu et al., 2016), suggesting heightened neuronal activity during withdrawal. The PVT projections to brain regions that regulate affective state implicate this increased activity in emotional components of withdrawal that drive continued or renewed drug seeking. However, the acute actions of MORs and the adaptive processes caused by chronic opioid exposure in PVT cells that project to these brain regions remain to be characterized.

The PVT is a heterogeneous population of neurons commonly divided into the anterior (aPVT) and posterior PVT (pPVT; Li and Kirouac, 2012; Gao et al., 2020; Rivera-Irizarry et al., 2023; Shima et al., 2023). Firing properties (Kolaj et al., 2012, 2014; McDevitt and Graziane, 2019), and gene expression profiles (Gao et al., 2020) further distinguish PVT neurons. Despite this heterogeneity, there is a uniformly dense expression of opioid receptors (Eacret et al., 2023; Hou et al., 2023). The aPVT in particular sends strong projections to limbic circuits implicated in opioid tolerance and withdrawal, including the nucleus accumbens and amygdala, and has been shown to have higher MOR expression relative to other thalamic regions (Chang et al., 2019; Hou et al., 2023). The PVT projection to the nucleus accumbens (NAc) has been specifically examined with respect to opioid use disorder as projections from the PVT to the NAc mediate active avoidance and gate the expression of opioid withdrawal behaviors (Zhu et al., 2016; Dong et al., 2020; Ma et al., 2021; Kanai et al., 2022; McDevitt et al., 2024). Other key projections from the PVT include the amygdala (AM) and medial prefrontal cortex (mPFC; Li and Kirouac, 2008; Gao et al., 2020), which contribute to the incubation of drug craving (Pickens et al., 2011). The projection from aPVT to amygdala encodes emotional valence (Barson et al., 2020; Kirouac, 2015; O’Neill et al., 2023; Penzo et al., 2015; Piantadosi et al., 2024), and projections to the basolateral amygdala (BLA) have been shown to modulate neuropathic pain and emotional anxiety (Tang et al., 2024). The mPFC is known to play a role in the preoccupation phase of opioid addiction (Koob and Volkow, 2016), and increased activity in the projections from the PVT to the mPFC have been shown to increase arousal and fear memory retrieval (Huong et al., 2006; Padilla-Coreano et al., 2012).

Despite the recognized importance of these projection-defined circuits, the acute activation of MORs and the adaptive processes resulting from chronic morphine treatment in projection-defined neurons of the PVT have not been fully characterized. Understanding the projection-specific acute and chronic actions of opioids is critical for elucidating the neural mechanisms underlying distinct components of opioid tolerance and withdrawal.

The present investigation examines the acute and chronic activation of MORs on aPVT neurons and the heterogeneity of these effects on cells that project to the NAc, amygdala, or mPFC. Acutely, aPVT neurons projecting to the amygdala were more sensitive than neurons projecting to the NAc and mPFC. Following chronic morphine treatment, neurons that projected to the amygdala and NAc developed increased receptor-dependent tolerance to morphine. The results indicate that chronic morphine treatment results in cellular adaptations in the neurons that project to three brain areas critically implicated in opioid use disorder.

## Materials and Methods

### Drugs

Morphine sulfate was obtained from the National Institute on Drug Abuse, Neuroscience Center. [Met^5^]-enkephalin (ME), bestatin, and thiorphan were acquired from Sigma-Aldrich. (*RS*)-Baclofen was purchased from Tocris. Stock solutions of ME and baclofen were dissolved in water, diluted to the appropriate concentration in artificial cerebrospinal fluid (ACSF), and applied by superfusion.

### Animals

Rats of both sexes between 5 and 8 weeks old were used for all experiments. Wild-type Sprague Dawley rats were obtained from Charles River Laboratories and maintained using approved breeding and husbandry procedures. MOR-cre rats were obtained from NIH (Bossert et al., 2023) and crossed with a floxed-tdTomato reporter rat (Igarashi et al., 2016), and offspring positive for both transgenes were provided by Dr M. Wolf at OHSU. All protocols and experiments were conducted in accordance with National Institutes of Health guidelines and with approval from the Institutional Animal Care and Use Committee of Oregon Health & Science University.

### Viral injections

Animals [postnatal day (P) 23–26] were anesthetized with 4% isoflurane and placed in a stereotaxic frame. Anesthesia was maintained with isoflurane (2.5%) and confirmed periodically by a lack of toe pinch response for microinjection of viral vectors. Injections of a retrograde adeno-associated virus (AAV) encoding green fluorescent protein (AAVrg-CAG-GFP) was carried out with the following coordinates (from bregma). (1) The amygdala (AM, anteroposterior: −2.5 mm, mediolateral: ± 3 mm, dorsoventral: −6.95 mm. (2) The nucleus accumbens (NAc) anteroposterior: +1.7 mm, mediolateral: ±0.7 mm, dorsoventral: −7 mm. (3) Medial prefrontal cortex (mPFC anteroposterior: +1.8 mm, mediolateral: ±0.5 mm, dorsoventral: −4.25 mm). A total of 200 nl of each virus was injected at 1 nl/s bilaterally in all regions. Electrophysiology experiments were carried out at least 2 weeks after injection.

### Chronic opioid treatment

Rats were treated with morphine sulfate continuously released from osmotic pumps as described previously (Quillinan et al., 2011). Osmotic pumps (2 ML1; Alzet) were filled with the required concentration of morphine (in water) to deliver 80 mg/kg/d at 10 µl/h for up to 7 d. The dose was chosen to induce the maximum amount of tolerance over the relatively short duration of application. For pump implantation, rats were anesthetized with 4% isoflurane, and anesthesia was maintained with 2.5% isoflurane. An incision was made in the midscapular region and osmotic pumps were implanted subcutaneously. The incision was then closed with 4–5 stainless steel wound closure clips (Stoelting; #59027), and animals were monitored to ensure stable healing. Pumps remained until animals were used for experiments 6 or 7 d later.

### Ex vivo slice preparation

Rats were deeply anesthetized using isoflurane and killed by cardiac percussion. Brains were excised, trimmed, and mounted adjacent to a 3% agar block (caudal edge) for slicing with a vibratome (VT 1200S; Leica). Horizontal PVT slices (272 mm) were prepared in warmed (∼34°C) and oxygenated (95% O_2_/5% CO_2_) ACSF containing the following (in mM): 126 NaCl, 2.5 KCl, 1.2 MgCl_2_, 2.4 CaCl_2_, 1.2 NaH_2_PO_4_, 21.4 NaHCO_3_, 11.1 d-glucose, and 10 µM MK-801 (to prevent NMDA-mediated excitotoxicity). Slices were allowed to recover in oxygenated ACSF at 34°C containing 10 µM MK-801 for ≥30 min and stored in oxygenated ACSF at 34°C until use. In experiments to assess tolerance and withdrawal, protocols were identical to those above with the addition of morphine hydrochloride (1 µM) to ACSF during extraction, slicing, recovery, and experiments.

### Electrophysiology

Slices were transferred to the recording chamber and continuously superfused with ACSF (1.5–2 ml/min, at 34°C). Recording pipettes (World Precision Instruments) with a resistance of 2–3 MΩ were filled with an internal solution containing the following (in mM): 100 potassium methanesulfonate, 20 NaCl, 1.5 MgCl2, 5 HEPES(K), 2 BAPTA (4K), 2 Mg-ATP, 0.3 NaGTP, adjusted to pH 7.35, and 275–280 mOsM. Whole-cell recordings from aPVT neurons were obtained using an Axopatch 1D amplifier (Axon Instruments) in voltage-clamp mode (*V*_hold _= −70 mV). Data was collected at 20 kHz and filtered at 10 kHz using AxoGraph X, and recordings were continuously monitored using PowerLab (Chart version 5.4.2; ADInstruments). Membrane resistance, capacitance, and series resistance were determined using the average of 20 5 mV pulses following break-in and were continuously monitored and checked upon termination of recordings. Cells were excluded if the series resistance at break-in was >20 MΩ, and experiments were terminated if the series resistance increased by >20% during recordings. Opioid currents were determined by the difference in the initial holding current and that induced by opioid agonists. Experiments that determined the current induced by morphine after chronic exposure were determined by the inward current induced by naloxone in the continued presence of morphine.

### Quantification of MOR-positive neurons

Rats expressing iCre recombinase under control of the endogenous *Oprm1* promoter were crossed with a floxed-tdTomato reporter rat to generate rats with tdTomato expression restricted to MOR expressing cells (Bossert et al., 2023; Igarashi et al., 2016). Slices for immunolabeling were generated using identical procedures as those for electrophysiology experiments. Following recovery, slices were fixed in 4% PFA in PBS for 1 h at room temperature. Fixed slices were blocked and permeabilized using PBS containing 0.5% Triton X-100 and 10% normal goat serum for 1 h at room temperature. Immunolabeling of NeuN (Millipore MAB377, 1:500) was conducted overnight at 4°C in PBS containing 0.1% Triton X-100 and 5% normal goat serum. Slices were washed three times for 20 min each in PBS at room temperature before secondary labeling with Goat anti-Mouse Alexa Fluor Plus 647 (Invitrogen A32728, 1:500) for 1 h at room temperature. Slices were then washed in PBS five times for ≥ 30 min each before being mounted on slides using Fluoromount-G (SouthernBiotech). Slides were cured overnight at room temperature and imaged the following day.

Imaging was conducted on a Zeiss LSM 710 controlled by Zeiss Zen software. An initial 10× image was taken to locate and define the aPVT using its position relative to the third ventricle. For quantification, tdTomato and NeuN signals were acquired sequentially to create *Z*-stacks (425 µm × 425 µm × 10 µm in *x*/*y*/*z*) using a 20× objective and large (∼2.5 AU) pinhole with identical laser and detection settings for all images. Images were denoised using Zen and maximum-intensity-projections were generated and tdTomato- and NeuN-positive cells were manually identified using individual channel images and the annotation function in Zen. Cells were then classified as positive for both NeuN and tdTomato using the independent designations and confirmed to be the same cell by shared morphology. The image presented is a representative maximum intensity projection used for quantification.

### Two-photon imaging

Two-photon imaging of cells expressing the AAV_retro_-GFP construct was carried out on a custom-built microscope using an Olympus BX51W1 upright platform and an Olympus 60×/1.0 NA water immersion LUMFI objective. Excitation was achieved using a Chameleon Ti:sapphire tunable laser at 910 nm. Image acquisition was conducted using ScanImage software (Pologruto et al., 2003). *Z*-stacks containing representative GFP-positive cells were generated at 2 µm steps and maximum intensity projections are shown.

### Data analysis

Sample sizes were not predetermined. For all experiments, 2–5 animals were used to obtain at least five technical replicates (cells) per group. Analysis was performed in AxoGraph X and statistics in GraphPad Prism 9. Values are presented as mean ± SEM or mean ± 95% confidence intervals as indicated in figure legends. Statistical comparisons were made using unpaired *t* test, Kruskal–Wallis, one-way or two-way ANOVA, as well as multiple comparison-adjusted Tukey's post hoc tests, Dunnett T3, and Šídák's test, as appropriate. For all experiments, *p* < 0.05 was used to define statistical significance.

## Results

### Opioid receptors in the aPVT

**Figure 1.**
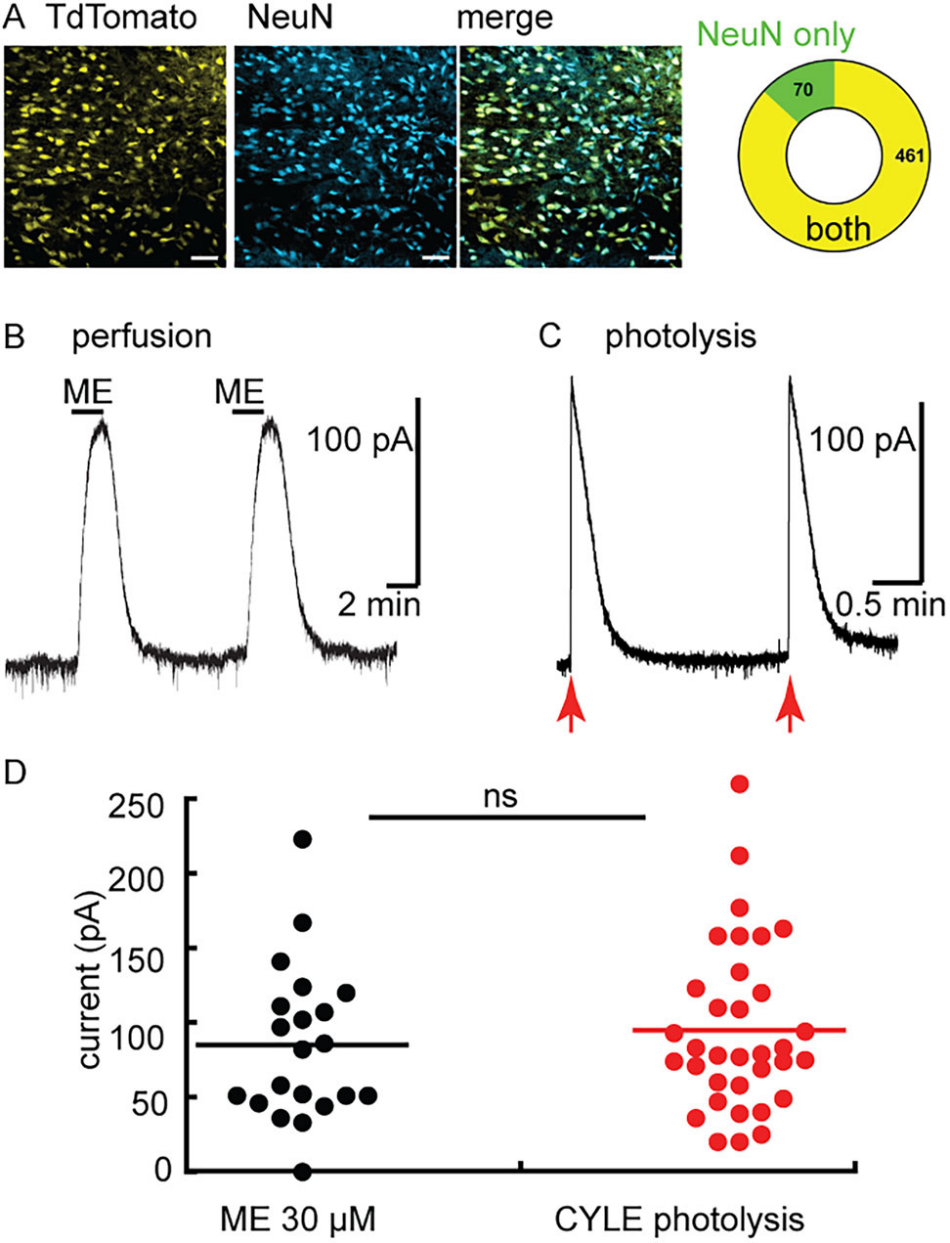
Dense expression of functional opioid receptors in the aPVT. ***A***, Representative images of NeuN immunolabeling and tdTomato expression in rats conditionally expressing tdTomato in MOR-positive cells. Left, Cre-dependent tdTomato cells; middle, immunofluorescence for NeuN indicating all neurons; and right, merged image of the two channels. Scale bar, 50 µm. Far right, Quantification indicating the number of cells that expressed both tdTomato and NeuN and cells that were stained with NeuN but did not express the tdTomato reporter (*n* = 531 cells/6 slices/2 animals). ***B***, Example recording showing the outward current induced by perfusion of ME (10 µM). ***C***, Example recording showing the outward current induced by photoactivation of CYLE. Arrows indicate the timing of a UV flash (50 ms, 5 mW). ***D***, Summarized results showing the outward current induced by perfusion of ME and photoactivation of CYLE (7 cells, 3 animals). The results indicate that activation of opioid receptors in the majority of neurons in the aPVT induced an outward current. Neurons in the aPVT express MORs. Supported by Extended Data Figure 1-1.

There is a high density of neurons that express MOR in the PVT (Mansour et al., 1994; Arvidsson et al., 1995; Le Merrer et al., 2009). The percentage of aPVT neurons that express MOR was determined by quantifying the coexpression of tdTomato with expression restricted to *Oprm1* positive cells (see methods) and NeuN immunolabeling. The results show that the vast majority (∼87%) of aPVT neurons coexpressed tdTomato and NeuN, though a small population of NeuN-positive cells that did not express tdTomato were observed (*n* = 531 cells/6 slices/2 animals; Fig. 1*A*). The MOR-mediated component of outward currents induced by ME was examined using whole-cell recording from the same *Oprm1*-Cre:tdTomato reporter animals (Extended Data Fig. 1-1). Application of ME (10 µM) induced an outward current (93.0 ± 8.9 pA, *n* = 6) that was blocked by coapplication of the MOR-selective antagonist, CTAP (1 µM; 8.9 ± 3.9 pA, 5 cells from 3 animals; *p* = 0.0003, unpaired *t* test). In experiments with wild-type animals, the application of ME (10 µM) or the photoactivation of caged [Leu]5-enkephalin (CYLE; Banghart and Sabatini, 2012) induced outward currents that ranged from 20 to 240 pA (ME 10 µM 84.9 ± 11.3 pA, 21 cells from 9 animals; CYLE 94.9 ± 9.6 pA, 35 cells from 13 animals, *p* = 0.5 unpaired *t* test) in amplitude (Fig. 1*B–D*). Thus, the majority of aPVT neurons express MORs that induce an outward current when activated.

10.1523/ENEURO.0249-24.2025.f1-1 Figure 1-1 Neurons in the aPVT express MORs. Left, a recording showing that the outward current induced by ME (10 µM) is reduced with prior application of CTAP (1 µM. Subsequent application of baclofen (10 µM) induced an outward current (108±16.3 pA, n=6). Right, Summarized results showing the current induced by ME (10 µM, 93±13.6 pA) prior to and the application of CTAP (1 µM, 8.9±3.9 pA, p=0.0013 paired T-Test, 6 cells, 3 animals). Download Figure 1-1, TIF file.

### An outwardly rectifying potassium conductance

**Figure 2.**
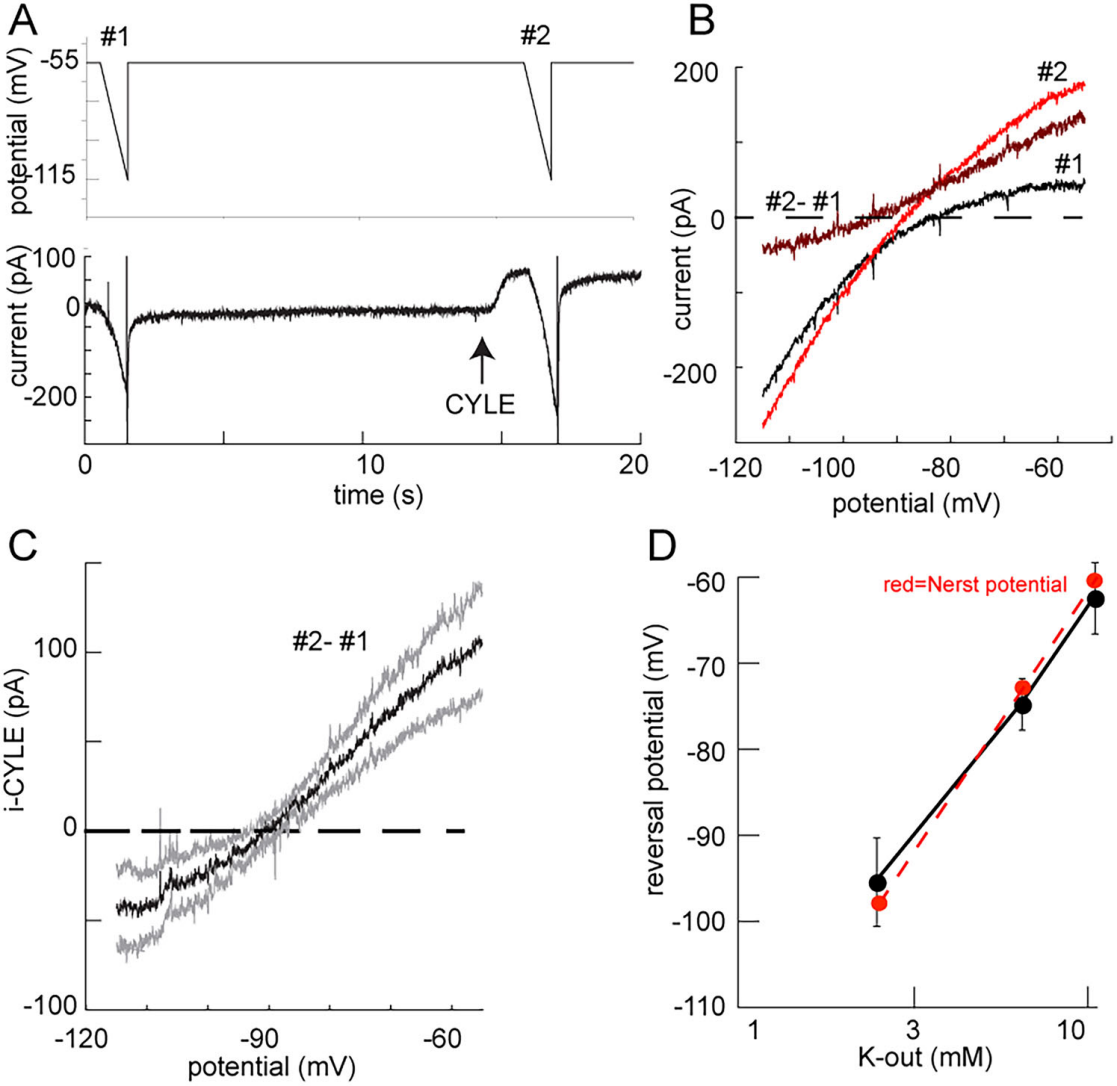
An outward rectifying potassium conductance is activated by opioid receptors. ***A***, An illustration of the protocol used to obtain the current/voltage plots. Two voltage ramps were applied and the currents induced during the ramps were measured before (#1) and following (#2) the photoactivation of CYLE. ***B***, Currents were recorded during voltage ramps. The current prior to photoactivation (#1) was subtracted from that following photoactivation of CYLE (#2) to obtain the isolated opioid-induced current. ***C***, Summarized results of the CYLE-induced current–voltage plot (dark line, mean; gray lines, 95% CL; 6 cells, 5 animals). ***D***, Summarized results (black line) showing the change in reversal potential in experiments using different concentrations of extracellular potassium (2.5 mM: −95.5 ± 5.1 mV; 6.5 mM −74.8 ± 3.0; 10.5 mM −62.5 ± 4.1 mV; *n* = 6, 5 animals). Red dashed line is the calculated Nernst potential in 2.5 K out is −98 mV, –6.5 K out is 72 mV, 10.5 K out is −60 mV). An outwardly rectifying potassium conductance. Supported by Extended Data Figure 2-1. Barium blocks the opioid currents. Supported by Extended Data Figure 2-2. ML297 augments opioid-induced potassium currents. Supported by Extended Data Figure 2-3.

The conductance induced by MOR activation was examined by the construction of current/voltage plots. The currents induced by ramp potentials from −55 to −125 mV were measured before and following photoactivation of caged [Leu]^5^enkephalin (CYLE; Fig. 2). A solution containing CYLE (50 µM) was perfused for a minimum of 5 min. Two 1 s ramp potentials from −55 to −125 mV were made 15 s apart in CYLE-containing ACSF. Before the second ramp, a 50 ms pulse of UV light (356 nm) was applied to photoactivate CYLE (Fig. 2*A*). Photoactivation resulted in an outward current measured at −55 mV in all cells tested (94.9 ± 9.6 pA, *n* = 35 cells from 13 animals; Fig. 1*D*). The current activated by CYLE during the potential ramp following photo activation was subtracted from the control ramp to obtain the CYLE-induced current (Fig. 2*B*,*C*). The opioid current induced by CYLE uncaging reversed at the potassium equilibrium potential (−97.8 mV, measured −95.5 ± 5.1 mV, *n* = 6 from 5 animals). Unexpectedly however, the opioid current measured at potentials more negative than the potassium equilibrium potential did not increase in amplitude as expected from an inwardly rectifying potassium conductance (G-protein-gated inwardly rectifying potassium conductance, GIRK). Similar results were found using voltage steps (10 mV) made from −45 to −125 mV with the current at each step measured in the absence and presence of ME (10 µM; Extended Data Fig. 2-1).

10.1523/ENEURO.0249-24.2025.f2-1 Figure 2-1 The outwardly rectifying potassium conductance measured with a voltage step protocol. A) The currents induced by voltage steps in the absence and presence of ME (10 µM). Voltage steps were made from -45 mV to -125 mV in 10 mV increments. B) The current voltage plots of the experiment illustrated in A. the ME current was determined by subtracting the control currents from those in the presence of ME. Download Figure 2-1, TIF file.

10.1523/ENEURO.0249-24.2025.f2-2 Figure 2-2 Barium blocks the opioid current induced by photoactivation of CYLE. A) The current measured during a ramp potential from -55 mV to -125 mV in the absence and presence of BaCl (1 mM). Arrow indicated the point of photoactivation of CYLE (50 ms). The addition of BaCl induced an inward current and blocked the outward current induced by CYLE. B) Summary of experiments with BaCl. The conductance induced by CYLE was blocked in the presence of BaCl (red). The dark lines indicate the mean current and the faint lines are the 95% CL (8 cells, 7 animals). Download Figure 2-2, TIF file.

**Figure 3.**
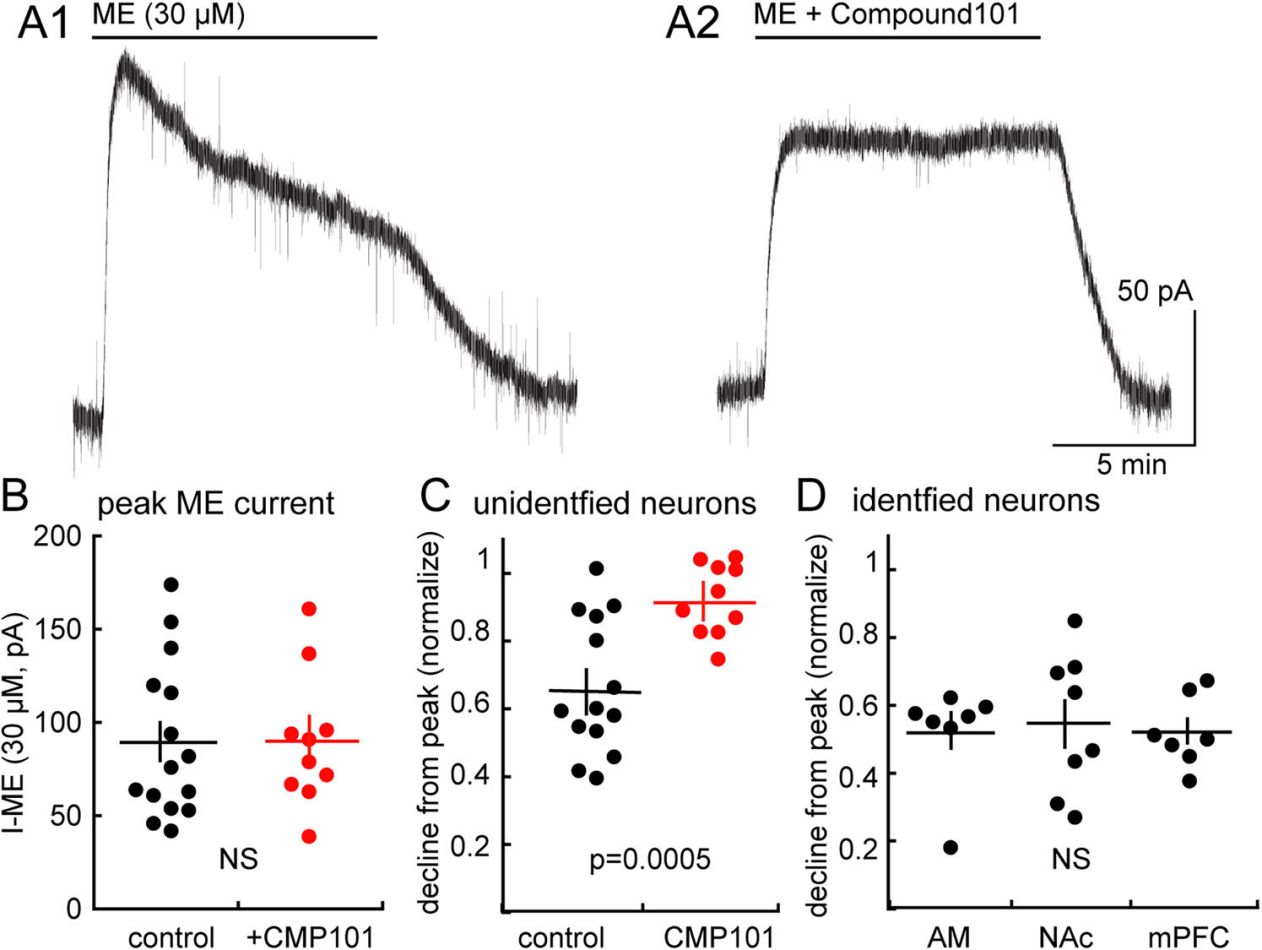
Desensitization of the opioid conductance is phosphorylation dependent. ***A***, Example currents induced by ME (30 µM) applied for 10 min in the absence (***A1***) or following incubation with (***A2***) compound 101 (10 µM, 1 h). Note the decline in current (acute desensitization) in ***A1*** that is abolished following compound 101 treatment (***A2***). ***B***, Summarized results showing that the peak current induced by ME (30 µM) was not different in control (88.9 ± 10.8 pA) or compound 101 treated (89.9 ± 11.3 pA) slices (control *n* = 15 cells from 11 animals, CMP101 *n* = 10 cells from 7 animals). ***C***, Summarized results of the decline in the ME current in control (black, 65.4 ± 5.3%) and that in slices treated with compound 101 (red, 91.35 ± 0.03%; *n* as above). ***D***, Results obtained with recordings from neurons with projections to the amygdala (AM), nucleus accumbens (NAc), and medial-prefrontal cortex (mPFC).

10.1523/ENEURO.0249-24.2025.f2-3 Figure 2-3 The potassium conductance is augmented in the presence of ML297 (10 µM). A) Example experiment illustrating the increase in CYLE induced current in the presence of ML297. B) The current voltage plot of the CYLE current obtained by subtracting the current illustrated in A) #1 from the current #2. The CYLE induced current was increased at all potentials. C) A summary of the current induced by CYLE in the presence of ML297 (from B #2). D) A summary of the ML297 sensitive current. The voltage dependence of the ML297 sensitive current (Part B #2-#1) is the same as that of the whole-cell current illustrated in part C. ML297 increased the conductance at all voltages though the current still rectified outwardly. (11 cells, 9 animals).  Download Figure 2-3, TIF file.

To further characterize the CYLE-induced outward current, the reversal potential of the current was measured in separate experiments where the potassium concentration in the extracellular solution was changed from 2.5 mM to 6.5 and 10.5 mM (Fig. 2*D*). The reversal potential of the CYLE-induced current measured in each concentration of potassium was −95.5 ± 5.1 mV (in 2.5 mM), −74.8 ± 3.0 (in 6.5 mM), and −62.5 ± 4.1 mV (in 10.5 mM; *n* = 6 from 5 animals). The predicted potentials based on the Nernst equation for a potassium and the observed reversal potentials were plotted as a function of the extracellular potassium indicating that the opioid current aligns well with the activation of a potassium conductance (Fig. 2*D*). The application of BaCl_2_ (1 mM) blocked the opioid current (Extended Data Fig. 2-2), further indicating an increase in potassium conductance. Because of the lack of inward rectification, it was not clear that the conductance was mediated by G-protein-gated inwardly rectifying potassium (GIRK) channels. The GIRK1 activator, ML297, was examined to determine if GIRK channels contributed to the conductance (Wydeven. et al., 2014). Application of ML297 (10 µM) did not affect the resting conductance but increased the outward opioid current suggesting that GIRK1 was involved (Extended Data Fig. 2-3). In the presence of ML297, the overall shape of the current/voltage plot did not change at potentials negative to the potassium equilibrium potential (Extended Data Fig. 2-3). The increase in the opioid current induced by ML297 between −55 and −115 mV was measured by subtracting the opioid-sensitive current obtained in control conditions from that in the presence of ML297 (Extended Data Fig. 2-3*D*). The result showed that the ML297-sensitive opioid current rectified outwardly similar to the current in control. Thus, this potassium conductance differed from the GPCR-dependent activation of GIRK observed in other neurons (Lüscher and Slesinger, 2010).

### Acute desensitization is phosphorylation dependent

Acute desensitization induced by a saturating concentration of an efficacious agonist, such as ME, varies among different neurons. For example, ME-induced desensitization in the LC is considerably larger than in neurons of the Kölliker–Fuse (Levitt and Williams, 2018). In the present study application of a saturating concentration of ME (30 µM) resulted in a peak outward current that declined to 65.4 ± 5.3% of the peak in 10 min (*n* = 15 cells from 11 animals; Fig. 3*C*). Acute desensitization is known to be dependent on phosphorylation of the C terminus of MOR by G-protein kinase (GRK; Lowe et al., 2015; Leff et al., 2020). The role of GRK in desensitization in aPVT cells was examined in slices incubated in the GIRK2/3 blocker, compound 101 (30 µM, 1 h). The amplitude of the initial current induced by ME (30 µM) was not significantly different in the absence (88.9 ± 10.8 pA) versus presence (89.9 ± 11.3 pA) of compound 101 (Fig. 3*B*; *p* = 0.4762; control: *n* = 15 cells from 5 animals; CMP101: *n* = 10 cells from 7 animals). In the presence of compound 101, however, desensitization induced by ME was completely blocked (remaining current at 10 min: 91.35 ± 0.03%; Fig. 3*A*,*C*). Thus, the desensitization of MORs in the aPVT is dependent on phosphorylation induced by GRK2/3.

### Opioid currents in projection-defined aPVT neurons

**Figure 4.**
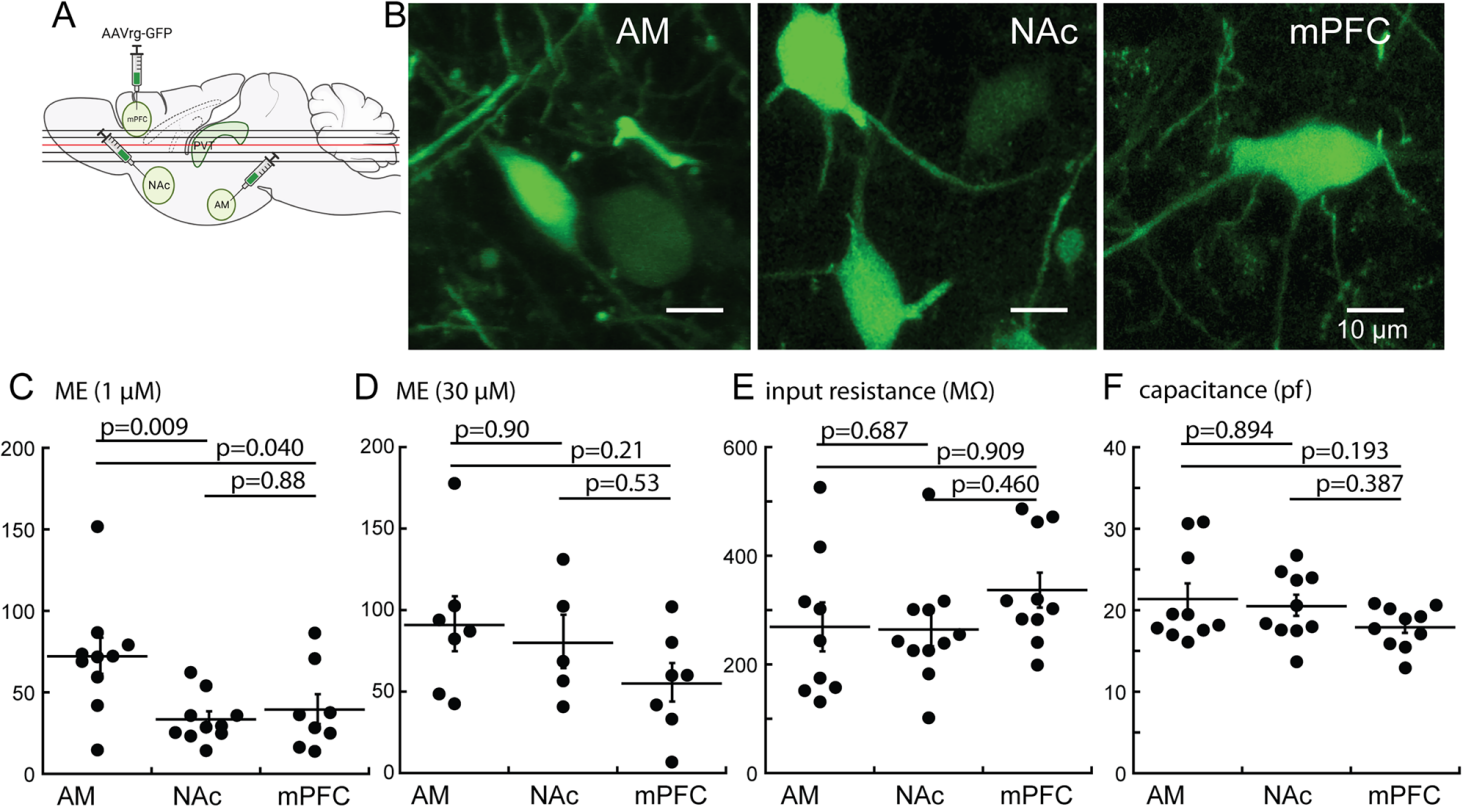
The sensitivity of neurons to ME (1 µM) varied among neurons with different projections. ***A***, Schematic illustrating the injection sites of retrograde AAV virus in three projection sites of the aPVT and dorsal/ventral planes where horizontal slices containing the aPVT were collected. ***B***, Examples of neurons expressing GFP from each of the projection areas (scale bar 10 µm). ***C***, Summary of the current induced by ME (1 µM) in neurons with different projections (AM: 72.2 ± 11.0 pA, NAc: 33.5 ± 4.6 pA, mPFC: 39.4 ± 9.2 pA; held at −70 mV, *n*_AM_ = 10 from 5 animals, *n*_NAc_ = 10 from 3 animals, *n*_mPFC_ = 8 from 3 animals). The current was largest in neurons that projected to the amygdala (*p*_AM_ vs _NAc_ = 0.0088, *p*_AM_ vs _mPFC_:0.0397, *p*_NAc_ vs _mPFC_: 0.88, Tukey's test following one-way ANOVA). ***D***, There was no difference in the currents induced by ME (30 µM) in projection neurons (AM: 90.8 ± 16.8 pA, NAc: 79.9 ± 16.3 pA, mPFC: 54.9 ± 11.82, pA, *n*_AM_ = 7 from 6 animals, *n*_NAc_ = 5 from 4 animals, *n*_mPFC_ = 7 from 4 animals; *p* > 0.05, One-way ANOVA). ***E***, ***F***, The input resistance (***E***; AM: 269.1 ± 45.0 pA, NAc: 264.4 ± 30.9 pA, mPFC: 336.9 ± 32 pA, *n*_AM_ = 10 from 5 animals, *n*_NAc_ = 11 from 3 animals, *n*_mPFC_ = 8 from 3 animals) and capacitance (***F***; AM: 21.4 ± 1.8 pf, NAc: 20.6 ± 1.2 pf, mPFC: 18.5 ± 0.9, pf, *n*_AM_ = 10 from 5 animals, *n*_NAc_ = 11 from 3 animals, n_mPFC_ = 8 from 3 animals) of neurons with different projections was not different (*p* > 0.05, one-way ANOVA), suggesting the difference in the current induced by ME was dependent on the coupling of MOR to the potassium conductance. Supported by Extended Data Figure 4-1.

The presence of opioid receptors on aPVT neurons with projections to the AM, NAc, or mPFC were examined by responses to ME. Retrograde AAV encoding GFP was microinjected into each area, and recordings were made from GFP-expressing cells (Fig. 4*A*,*B*). The currents induced by ME (1 µM) in recordings from neurons that projected to the AM were larger compared with those projecting to the NAc and mPFC (AM: 72.2 ± 11.0 pA, NAc: 33.5 ± 4.6 pA, mPFC: 39.4 ± 9.2 pA, *n*_AM_ = 10 cells from 5 animals, *n*_NAc_ = 10 cells from 3 animals, *n*_mPFC_ = 8 cells from 3 animals; Fig. 4*C*). A concentration response to ME indicated that the peak current was reached between 3 and 30 µm ME (Extended Data Fig. 4-1). The currents measured in projection-defined cells were not different when a saturating concentration of ME (30 µM) was applied, suggesting that neurons projecting to the AM were more sensitive than those that projected to the NAc or mPFC (Fig. 4*D*). The capacitance and resting membrane resistance were similar in each projection, indicating that the size and resting conductance of the neurons were not the determinants of the variable amplitude of the opioid current (Fig. 4*E*,*F*). Although the sensitivity of neurons varied between neurons with different projections, the extent of desensitization (decline from peak) was similar, indicating that the size of the current was not a determining factor in acute desensitization (Fig. 3*D*).

10.1523/ENEURO.0249-24.2025.f4-1 Figure 4-1 A concentration response curve to ME. The current amplitude was normalized to the capacitance of each neuron (current density, pA/µF). The plot indicates that ME applied at concentrations above 3 µM induce a maximum current. Download Figure 4-1, TIF file.

### Chronic morphine induces tolerance

**Figure 5.**
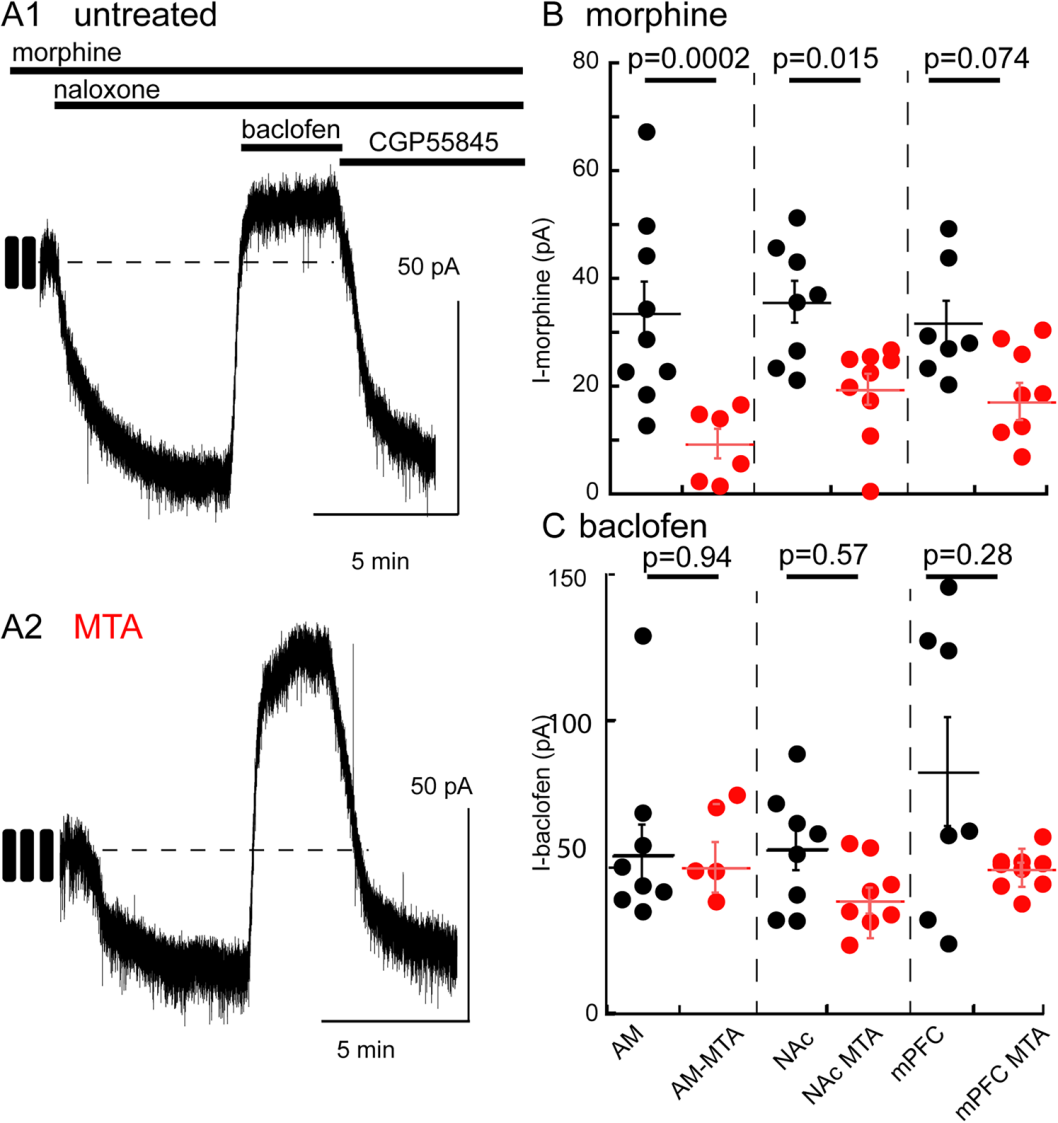
Chronic treatment of animals with morphine-induced tolerance in neurons that projected to the AM and NAc. ***A***, Example recordings of the protocol used to determine tolerance to morphine. Slices from untreated (top) and morphine-treated (bottom) animals were cut and maintained in the presence of morphine (1 µM). Recordings were made and naloxone (1 µM) was applied to reverse the morphine-induced current. This was followed by the application of baclofen (10 µM) and CGP55845 (300 nM) to reverse the baclofen-induced current. ***B***, Summarized results obtained from untreated (black) and morphine-treated (red) animals. Top, The morphine-dependent current in slices from morphine-treated animals was smaller in neurons projecting to the AM (Naive: 33.1 ± 5.7, MTA: 11.7 ± 2.7, *p* = 0.0065, Tukey's test following two-way ANOVA, *n*_Naive_ = 9 from 4 animals, *n*_MTA_ = 6 from 4 animals) and NAc (Naive: 36.7 ± 3.8, MTA: 19.2 ± 2.8, *p* = 0. 0.0177, Tukey's test following two-way ANOVA, *n*_Naive_ = 8 from 4 animals, *n*_MTA_ = 9 from 4 animals) but not those that projected to the mPFC (Naive: 34.3 ± 4.0, MTA: 18.6 ± 3.0, *p* = 0.0714, Tukey's test following two-way ANOVA, *n*_Naive_ = 7 from 7 animals, *n*_MTA _= 9 from 3 animals). Bottom, The current induced by baclofen was not different in slices from untreated and morphine-treated animals in all neurons. Supported by Extended Data Figure 5-1.

The effects of chronic opioid treatment were examined following treatment of animals with morphine for 6–7 d using osmotic minipumps (80 mg/kg/d). Receptor-dependent tolerance was investigated using a protocol where slices were prepared and maintained in a concentration of morphine (1 µM) that approximated the brain concentration induced by the osmotic minipump delivery (Quillinan et al., 2011). Slices from untreated animals were also prepared and maintained in the presence of morphine (1 µM). Recordings were made from neurons with identified projections. A steady baseline of 2–5 min in the presence of morphine was obtained prior to the application of naloxone (1 µM). The magnitude of change in holding current induced by naloxone was determined by the difference in the baseline current averaged over 15 s preceding naloxone perfusion and the average current over 15 s measured 5 min following the application of naloxone (Fig. 5). Following the application of naloxone, the peak current (again averaged over a 15 s window centered around the peak) induced by a saturating concentration of baclofen (10 µM) was measured to examine nonspecific alterations the potassium current (Fig. 5). The morphine-dependent current in slices from morphine-treated animals (MTA) was significantly decreased relative to that in slices from untreated controls in AM- and NAc-projecting neurons; there was a trend in mPFC-projecting cells that did not reach statistical significance (AM-Naive: 33.1 ± 5.7, MTA: 11.7 ± 2.7, *p* = 0.0065, *n*_Naive_ = 9 from 4 animals, *n*_MTA_ = 6 from 4 animals; NAc-Naive: 36.7 ± 3.8, MTA: 19.2 ± 2.8, *p* = 0. 0.0177, *n*_Naive_ = 8 from 4 animals, *n*_MTA_ = 9 from 4 animals; mPFC-Naive: 34.3 ± 4.0, MTA: 18.6 ± 3.0, *p* = 0.0714, *n*_Naive_ = 7 from 7 animals, *n*_MTA_ = 9 from 3 animals, 2-way ANOVA with Tukey's test; Fig. 5*A*,*B*). The current induced by baclofen was not different in experiments in slices taken from untreated and MTA in any of the three populations (Fig. 5*C*; *p* = 0.2603, Kruskal–Wallis test) indicating that the tolerance to morphine was specific to morphine (homologous). Only mPFC-projecting aPVT neurons displayed an increase in membrane resistance following chronic morphine treatment (Naive: 248.9 ± 35.1 MΩ, MTA: 365.8 ± 35.1 MΩ, *p* = 0.0384, unpaired *t* test; Extended Data Fig. 5-1). No difference was seen in the membrane resistance of the other AM- or NAc-projecting neurons (AM: Naive: 295.2 ± 49.7 MΩ; MTA: 297.2 ± 53.8 MΩ; *p* = 0.5; NAc: Naive: 249 ± 27.6 MΩ; MTA**:** 298.3 ± 50.5 MΩ; *p* = 0.21, unpaired *t* tests). Additionally, no change was seen in the capacitance of following chronic morphine treatment (AM: Naive: 18.84 ± 1.39 pF; MTA: 17.9 ± 1.7 pF; *p* = 0.67; NAc: Naive: 19.8 ± 1.3 pF; MTA: 18.2 ± 1.5 pF; *p* = 0.44; mPFC: Naive: 22.6 ± 2.9 pF; MTA: 17.5 ± 1.4 pF; *p* = 0.11, unpaired *t* tests).

10.1523/ENEURO.0249-24.2025.f5-1 Figure 5-1 Chronic treatment of animals with morphine increased membrane resistance selectively in mPFC-projecting aPVT neurons. (A–C) Membrane resistance was significantly increased by morphine treatment only in neurons projecting to the mPFC (Naïve: 249.9 ±35.5 MΩ, MTA: 365.8 ±35.1 MΩ; p = 0.0384, t-test, n_Naïve_ = 7 cells from 7 animals, n_MTA_ = 9 cells from 3 animals) but not in neurons projecting to the AM (Naïve: 295.2 ±49.7 MΩ, MTA: 297.2 ±53.8 MΩ; p = 0.5, Mann-Whitney test, n_Naïve_ = 9 cells from 4 animals, n_MTA_ = 6 cells from 4 animals) or NAc (Naïve: 249.2 ±27.6 MΩ, MTA: 298.3 ±50.5 MΩ; p = 0.21, t-test, n_Naïve_ = 8 cells from 4 animals, n_MTA_ = 9 cells from 4 animals). (D–F) Capacitance did not differ significantly between naïve and morphine-treated animals in any of the projection populations (AM: Naïve 18.8 ±1.4 pF, MTA 17.9 ±1.7 pF, p = 0.67; NAc: Naïve 19.77 ± 1.3 pF, MTA 18.2 ±1.5 pF, p = 0.22; mPFC: Naïve 22.6 ±2.91 pF, MTA 17.5 ±1.4 pF, p = 0.11; t-test, data are shown as mean ± SEM). Download Figure 5-1, TIF file.

## Discussion

This study characterized the acute and chronic actions of opioids on neurons of the anterior paraventricular thalamus (aPVT), highlighting projection-specific differences in both sensitivity and adaptations induced by chronic treatment of animals with morphine. Using a conditional transgenic reporter rat to identify MOR expressing neurons, we observed that most (∼90%) aPVT neurons express µ-opioid receptors (MORs). The application of opioids induced an outward current mediated by an increase in potassium conductance that was antagonized by CTAP, a specific MOR antagonist. The outward potassium current was unique in that the current/voltage relationship lacked the typical inward rectification of GIRK channels but was augmented by a positive modulator of GIRK1 channels, ML297. Acute desensitization of the current induced by opioids was dependent on phosphorylation by G-protein receptor kinase 2/3 (GRK2/3), as determined by its blockade by the GRK2/3 inhibitor compound 101. Desensitization as measured by the decline in the peak outward current induced by ME (30 µM, 10 min) measured in the aPVT exhibited an intermediate level (65 ± 5%) relative to that found in the locus ceruleus where acute desensitization is prominent (56 ± 3%; Levitt and Williams, 2018) and the Kölliker–Fuse nucleus where desensitization is limited (72 ± 14%; Levitt and Williams, 2018). Following chronic morphine treatment, a reduction in the current induced by morphine was observed in neurons that project to the AM and NAc. An important limitation of this study is the small sample size and high variance observed within some experimental groups, which could potentially mask underlying differences. Thus, interpretation must be tempered, acknowledging the possibility that larger cohorts or reduced variance might reveal additional meaningful differences. Taken together, however, the results show that the aPVT exhibits both acute responses to MOR activation and adaptive changes following chronic morphine treatment.

Neurons in the PVT contribute to multiple behaviors dependent on the projection target, which can drive distinct aspects of affective or defensive states. For example, the PVT to central AM pathway drives conditioned freezing responses, whereas the PVT to NAc pathway signals active avoidance events (Choi et al., 2010; Oleson et al., 2012; Li et al., 2013; Bravo-Rivera et al., 2014; Penzo et al., 2015; Ramirez et al., 2015; Fadok et al., 2017). In addition, the aPVT and pPVT send nonoverlapping projections to areas including the NAc, amygdala, and mPFC, further highlighting the heterogeneity of the PVT (Gao et al., 2020; Shima et al., 2023). The results from the present study indicate that AM-projecting neurons have a larger potassium-mediated conductance induced by subsaturating ME concentrations when compared with those projecting to the NAc and mPFC. Given that the cell capacitance and input resistance were similar between neurons in each of the projection areas, it is unlikely that cell size and resting conductance play a role in the different amplitudes of the current induced by ME. The response to a saturating concentration of ME was not significantly different between projections, indicating that AM-projecting neurons are more sensitive to opioids compared with NAc- and mPFC-projecting neurons. Varied coupling of MORs to the potassium conductance or an increased expression of MORs could underlie the difference in current amplitudes at subsaturating concentrations. Functionally, the sensitivity of AM-projecting neurons to opioids may influence emotional processing during opioid use, potentially attenuating affective pain processing. In contrast, the increased receptor-dependent tolerance observed following morphine treatment in neurons projecting to the NAc and mPFC may facilitate motivated behavior toward drug seeking and altered cognitive processes, respectively. Projection-specific adaptations could differentially affect neural circuits within a single brain region involved in opioid use disorder, likely underlying to its multifaceted behavioral manifestations.

The outward current was mediated by a potassium conductance that was distinctive in that the current/voltage (*I*/*V*) relationship lacked a typical inward rectification. A previous study indicated that a MOR-mediated hyperpolarization was not solely determined by GIRKs as indicated by the inability of tertiapin-Q, a GIRK blocker, to completely block the opioid current or the MOR-mediated hyperpolarization (Hou et al., 2023). The results from that study along with the current/voltage plots of the opioid current made in the present investigation suggest that the underlying conductance is not dependent solely on GIRK channels. Single-cell RNA sequencing revealed expression of the GIRK subunits *KCNJ3*, *KCNJ6*, *KCNJ9*, and *KCNJ5* in the aPVT (Gao et al., 2020; Allen Brain Atlas). The fact that ML297 increased the amplitude of the opioid current suggests that GIRK1 may be involved; however, ML297 did not induce an increase in inward rectification or alter the shape of the current/voltage plot. This suggests a complex interaction between GIRK channels and likely other potassium channels affecting neuronal excitability. The identity of the potassium conductance activated by opioids remains a question. One possibility is the two-pore domain potassium (K2P) channel family. Knockout mice lacking TREK-1 channels were significantly less sensitive to morphine, indicating that opioids could act through this channel (Devilliers et al., 2013). In addition, morphine caused an outward current in COS cells expressing TREK-1 channels, and the current–voltage plot was similar to that found in the present study (Devilliers et al., 2013). Thus, the conductance underlying the outward current in the PVT may involve K2P channels, potentially in conjunction with GIRK channels, reflecting a complex interplay that affects neuronal excitability.

Projections to the NAc, mPFC, and AM are likely all key areas in both the positive and negative aspects of acute and chronic actions of opioids. The symptoms of acute withdrawal are thought to contribute to opioid use disorder in that continued drug seeking is maintained to avoid aversive states (Koob and Volkow, 2016; Koob, 2020). The connections between the PVT and limbic structures, such as the amygdala and NAc, play critical roles in processing such negative states. Naloxone-induced withdrawal results in a substantial increase in c-*fos* expression in the PVT as well as potentiated signaling from PVT cells on to D_2_ receptor expressing cells in the NAc (Zhu et al., 2016). The role of pre- and postsynaptic adaptations in altered PVT activity have yet to be fully explored; however, it is likely that both contribute to such effects. The concept of hyperkatifeia, or increased sensitivity to emotional distress, is one well-studied aspect of the motivational aspects of continued opioid use (Koob, 2020). The aPVT is poised to encode this increased sensitivity through its influence on limbic circuits, reinforcing drug-seeking behavior as a means to alleviate negative emotional states following chronic drug use.

## Synthesis

Reviewing Editor: Michael Michaelides, NIDA-NIH Decisions are customarily a result of the Reviewing Editor and the peer reviewers coming together and discussing their recommendations until a consensus is reached. When revisions are invited, a fact-based synthesis statement explaining their decision and outlining what is needed to prepare a revision will be listed below. The following reviewer(s) agreed to reveal their identity: James Otis, Brenton Laing, Adam Caccavano.

Reviewer 2

Author Response to Reviewer Major Concern about Figure 1: It is true that there are many opioid positive processes of both axonal and dendritic origin. Although the RNAscope will identify neurons that can express opioid receptor, the NAI594 actually identifies opioid receptors. We have used NAI504 to identify neurons expressing the opioid receptor in many preparations, made recordings from those neurons and measured functional responses to opioids (Arttamangkul et al., 2021). Thus, it is a reliable way to identify neurons that express receptors. We have modified figure one to show that the greatest percentage of neurons in the aPVT are sensitive to opioids. If this is still considered to be a serious problem, we can remove the figure with the NAI594. We also have preliminary results with the cross of the MOR-cre with a tdTomato reporter rat showing the dense number of neurons that are positive for cre expression (attached).

Reviewer Response: The authors have made no effort to quantify the opioid receptor data in Fig 1. Furthermore, the figure does not show the percentage of neurons that are sensitive to opioids as authors stated. Instead, the authors put a recording that was in previous Fig 2 into Fig 1, related to electrophysiological current responses to ME application.

Author Response to Reviewer Comment about Error Bars: The error bars have been added - Many journals now prefer the use of SD rather than SEM as it is a better representation of the distribution of the results. We prefer to use the SD.

Reviewer Response: The authors did not adequately consider my concern, and unfortunately data is being misrepresented within the manuscript. For example, the reviewer's simulation of individual data points from Fig 1 shows that SEM (~+/-11.1) is displayed. Yet, the text indicates that data is reported as SD (~ +/- 35.1). This is an issue for a majority of the figures.

----------------------------------------------------------------------------------------------------------------------------------------------

Reviewer 3

Summary: The authors report that neurons of the rat paraventricular thalamus (PVT) have some commonality (e.g. >90% show potassium conductance, a peak concentration of conductance that declines during exposure to saturating concentration). Using previously known information that PVT neurons can be segregated by projection targets (NAc, amygdala, and PFC), these authors report that the potassium current in neurons that project to amygdala is the largest. They report that the peak amplitude declines over time, which they and others in the literature term as desensitization, and that this decline can be blocked by co-application of compound 101. The authors report that amygdala and NAc were less sensitive to morphine in brains taken from mice who have been chronically administered morphine via implantable pump. Input-frequency testing indicated that NAc projecting PVT neurons exhibit greater number of action potentials, which they associate with withdrawal.

Overall this is an interesting manuscript with high level techniques that merge electrophysiological and imaging approaches with pharmacology of drug application. The observation reported here that compound 101 blocks desensitization appear to be a novel advance, as does project-region specific adaptation of neuronal activity following current injection. While these studies provide little information about the consequence of the mechanisms identified in terms of opioid use disorder pathology or withdrawal symptoms, they add to the body of literature for the total number of changes that occur during prolonged morphine exposure.

Major comments:

The statistical reporting could be substantially improved in the text or ideally by including a statistical table as described in eNeuro guidelines. Specific p-values are not consistently stated, degrees of freedom are not indicated, test-statistic values are not listed, and assessment of assumptions is not stated. Inclusion of this information would greatly increase the likelihood of future investigators building off of this data. Further, sometimes statistics appear in the legend (e.g. Figure 3) and sometimes they appear on the figure (e.g. Figure 5). A table will help improve consistency, reader access to the information, and enable more thorough assessment of the claim that NAc projecting neurons have a higher firing rate after chronic exposure, but not amygdala or PFC.

The claim that this naloxone application in brain slices is withdrawal is tenuous. All references to withdrawal should be restated as µOR antagonism effects using competitive antagonist naloxone. The data reported here are interesting and do not need to be overstated.

The authors make 8 key claims in this article, 4 of which require clarification.

Claim 2: The outward potassium current in neurons that projected to the amygdala was larger than those projecting to the nucleus accumbens and medial PFC

-Is the highest responder in the amygdala an outlier (as indicated by Grubbs outlier test or comparable)? If so, does the effect stand without the data from that cell?

Claim 6: Chronic morphine exposure decreases amygdala and NAc sensitivity to morphine greater than PFC

-Within structure differences appear to be tested, but between structure do not. If the authors want to make claims about between structure differences (e.g. NAc vs. PFC), then they should report the relevant statistical analysis to support this claim. Otherwise, they should only reference the within structure differences.

Claim 7: NAc projecting PVT neurons have a higher firing rate after chronic exposure, but not amygdala or pfc

-Higher response to a 100pA injection in NAc of morphine treated animals than untreated animals; not observed in mPFC or BLA. This matches the text of the abstract. However, line 382-384 state, "In recordings from neurons that projected to NAc and amygdala the number of action potentials induced by depolarizing current in the presence of morphine was larger in slices taken from morphine treated animals than untreated controls." From the figure, it is unclear if the mPFC results are significant or not. This is compounded by the lack of statistical reporting. The figure legend says that statistical significances are indicated by p-values, but there are no indications on the figure (even for NAc).

Claim 8: Separate projections from the PVT exhibit differential tolerance and withdrawal and therefore play different roles in development of OUD

All references to withdrawal should be restated as µOR antagonism effects. Withdrawal is an emergent state in living animals marked by well characterized behavioral symptoms

Minor comments:

1) The representative trace in figure 1C is not representative of the mean current that is calculated. It appears to be the largest responder.

2) Why do figure 1 and 5 refer to amygdala, but figure 6 refers to BLA?

3) Figure 2 does not have an X-axis or Y-axis label.

4) It is unclear why the peak amplitude of ME treatment is lower with compound 101? This effect is not observable in Lowe et al. 2015. This discrepancy should be highlighted or explained in the discussion.

----------------------------------------------------------------------------------------------------------------------------------------------------------------------

Reviewer 4

The study focuses on an important and interesting question, the response of opioids within the PVT, and employs a useful technique, retrograde labeling with subsequent electrophysiology and histology, to distinguish specific actions on subpopulations within the region. The findings of this study have the potential to advance the field and guide future studies. However, there are numerous methodological and presentation concerns detailed below that limit this objective. Most of the below comments could be addressed with further analysis and edits to the text and figures. Two highlighted concerns would require additional experiments that the authors should have already completed.

1) The electrophysiological and morphological characterization of the labeled aPVT subpopulations (Fig 1, 1-1) is lacking, with data presented only for membrane resistance, capacitance, and response to met-enkephalin. Several later conclusions concerning divergent responses of these subpopulations would be impacted with divergent electrophysiological properties such as a change in resting membrane, spike threshold, or spike properties. Intrinsic data is later presented with morphine onboard, but the baseline characterization of these cells is missing.

2) The internalization of receptors represents a nice experimental approach and analysis (Fig 4) but is missing an adequate control.

Results/Figures:

Fig 1B: Some counts/quantification of images needed. e.g. density of retrogradely labeled cells for each injection (B, top), and % of retrogradely labeled cells colocalized with MOR (B, bottom).

Fig 1B, bottom. needs scalebar

Fig 1C-D: need to specify the holding voltage

Fig 1-1. It would be a benefit to the field and some later conclusions in this study to present the full electrophysiological characterization of these retrogradely labeled aPVT subpopulations (RMP, firing characteristics, etc), either in plots (in addition to the capacitance and resistance as presented in 1.1), or in a supplemental table. While presented to some degree in 6-1, this is only with morphine onboard.

Fig 2-1. Need to specify n (cells and mice), and if sufficient n allows, to do quantification across cells, as done in Fig. 2, 2-2, and 2-3.

Fig 2-1B. Mark the axis (y = 0) to make a deviation from zero current clearer, as done in Fig. 2B-C. In contrast to Fig 2B, there appears to be stronger outward rectification - discuss more. Missing axis labels.

Fig. 2D. Plot could be represented clearer. Blue and red lines are meaningless, while thin black looks similar to thick black lines. It would be more instructive to present an experimental average and Nernst-predicted theoretical curve (for a given K-in).

Fig. 2-2 &2-3. Need to specify n (cells and mice) to obtain confidence intervals.

Fig. 2-3B-C. Keep on same scale

Lines 316-317: "... Thus, this opioid-sensitive potassium conductance differed from the activation of GIRK that has been observed in other neurons" it's unclear if referring to MOR-associated GIRKs or GIRKs in general, and in any case should have a citation to point the reader to.

Lines 325-326: "Although the peak amplitude of the ME-induced current varied between neurons with different projections..." this is of interest, why not show? Does saturating concentration follow same pattern as Fig. 1D? Could be of interest in relation to the comment above about any potential different densities of opioid receptors in these subpopulations.

Fig. 3B, D Suggestion: Put on same scale for comparison and make sure to have 1 (100%) with label

Fig. 3D. Specify from what projection subpopulations these cells were taken (same cells as B pooled together, or subset?)

Fig. 3. Small point but legend states SD while methods states data shown as mean {plus minus} SEM. Either is fine, just be consistent.

Line 331 typo: GRK not GIRK

Line 340 typo: 594 or 549

Fig. 4: Naïve is a meaningless comparison, in taking a ratio of baseline to itself (which results in 1 as shown and not zero as stated). Should not be included. A true control should be included, which would just be a vehicle treatment over the same time course, or additionally of interest, a non-saturating 1 µM dose as applied in Fig. 1.

Fig. 5B. In text it states the morphine current was normalized by baclofen response. Is this normalized here or morphine current?

Fig. 6 &6-1. Related to above comment about potential electrophysiological differences between these subpopulations in Fig. 1 - baseline differences in threshold and spiking properties could impact these measures with morphine/naloxone. As data are presented as untreated vs MTA within each subpopulation, this should be somewhat controlled. But still, without knowing what these baseline differences in cell properties are, this presents a caveat when interpreting observed differences here between these subpopulations.

Fig. 6D. do 2-way ANOVAs to backup stated conclusions.

Fig. 6-1. Specify statistical test - 2-way ANOVA?

Discussion:

Line 432-433: " Thus, the coupling of MORs to the potassium conductance is likely the determinant of the current amplitude." It is a possibility. But could be several other mechanisms. More opioid receptors, different potassium channels, etc.

Lines 449-450 "however, ML297 did not increase the current at potentials more negative than the potassium equilibrium potential." Unclear what the authors mean here. Fig. 2-3B shows a pretty clear increased inward current at voltages hyperpolarized to the reversal potential (V < -90mV) relative to control.

Lines 479-487 are surprising, and if correctly stated these data need to be shown in a figure of their own. The left two bars of Fig. 6B for each region essentially compare (acute morphine) to (chronic morphine), and intuitively show that chronic morphine treated cells are more excitable, presumably through desensitization/tolerance (though as stated above - this figure needs stats). But the statement here claims that chronic morphine treatment alone increases spontaneous activity relative to untreated (presumably without 1µM morphine in the bath)? This is a unintuitive result considering all the previous work in this manuscript showing that opioids activate a hyperpolarizing K+ current in these cells. I think it may be a misstatement and is instead referencing the data in Fig. 6 - in which case I would caution there is no true "untreated" condition here, as there is always morphine onboard.

Overall:

The paper mentions differences between anterior and posterior PVT, though never states why the present study has focused on the aPVT.

Results/Discussion subheadings and figure titles would benefit from succinct statements of the principal findings instead of statements of general topics (eg "withdrawal").

The several different methods to activate MORs warrants a little more description of the affinities of each drug (met/leu-enkephalin/morphine). As enkephalins have higher affinity to DORs vs MORs, important to know if DORs are expressed in aPVT.

---------------------------------------------------------------------------------------------------------------------------------------------------

Reviewer 5

In this manuscript, the authors report that the magnitudes of mu opioid receptor agonist responses in anterior PVT neurons are related to projection target. Specifically, outward currents in response to met-enkephalin were larger in amygdala-projecting neurons compared to those that project to the nucleus accumbens or medial prefrontal cortex. The authors provide evidence that these responses might be mediated by a combination of GIRK and other K+ currents. The authors also quantify acute opioid receptor desensitization and changes in opioid receptor and GABAB receptor function driven by repeated in vivo morphine treatment. Together these observations are potentially interesting, however some issues need to be addressed:

For the pharmacology experiments, it is not reported how the magnitudes of the drug responses (change in holding current) was quantified.

In figure 5, it looks like some of the comparisons would actually be statistically significant if there were a few more samples. The 7-8 presented here is the bare minimum, but given common levels of variance observed in electrophysiology, it is not unusual to need 10-12 neurons in order to power experiments appropriately.

Some of the datasets appear to violate the requirements for parametric statistical testing, yet only parametric tests were utilized. Please use non-parametric statistics where appropriate.

How do you know the ME and LE effects are MOR mediated? These peptides are not selective for MOR. One potential way to check that these responses are MOR mediated are to block or reverse the responses with a MOR selective antagonist, like CTOP or CTAP.

The authors indicate in some places that saturating concentrations of an agonist are used, for instance met-enk. How do they know this is a saturating concentration? Potency and efficacy vary between brain regions, so this would have to be demonstrated specifically in PVT.

Similarly, how do the authors know that 1 uM naloxone is sufficient to completely reverse the ongoing morphine actions? This seems like a potentially low concentration, given that 1 uM morphine is in the bath. And 1 uM of each of these drugs could have off target, non-MOR effects.

Were all neurons included, including those with no detectable opioid responses?

How were the neurons selected for analysis in Figure 4?

There are still no scale bars in the higher magnification images in Fig 1B.

The text seems to say that the theoretical Nernst-calculated K+ reversals potentials are included in Fig 2D, however this is not clear in the figure.

Number of samples is missing from some of the figures/figure captions.

The sources for some of the pharmacological agents are not listed in the Methods.

There are several grammatical issues throughout the manuscript. In some places this makes it difficult to follow.
