# Supplementary figures and images for: Tolerance in Thalamic Paraventricular Nucleus Neurons Following Chronic Treatment of Animals with Morphine
Source: eNeuro. 2025 Jun 6;12(6):ENEURO.0249-24.2025. doi: 10.1523/ENEURO.0249-24.2025 (PMC12177709; doi:10.1523/ENEURO.0249-24.2025)

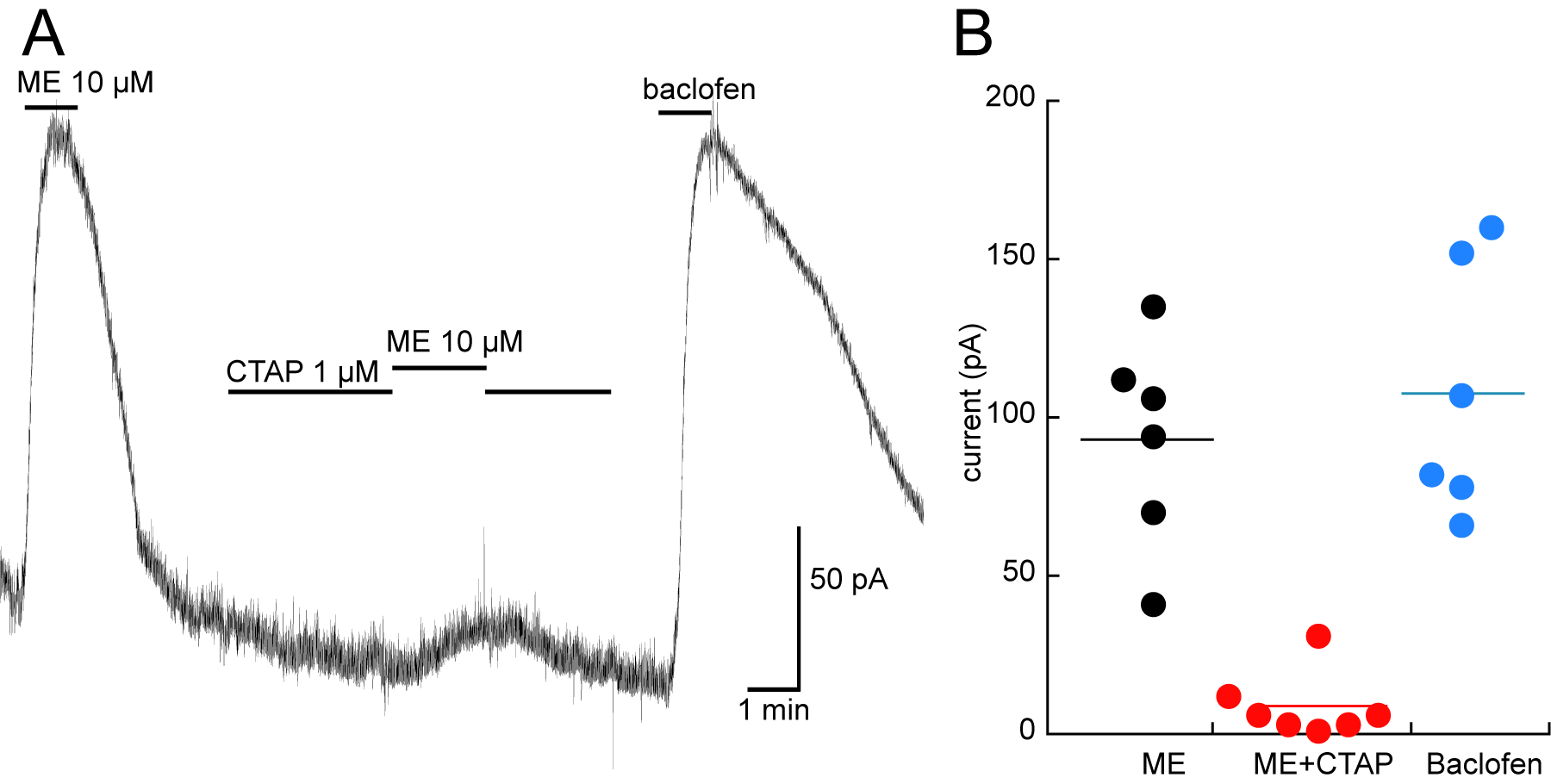

Supplement: Figure 1-1 — Neurons in the aPVT express MORs. Left, a recording showing that the outward current induced by ME (10 µM) is reduced with prior application of CTAP (1 µM. Subsequent application of baclofen (10 µM) induced an outward current (108±16.3 pA, n=6). Right, Summarized results showing the current induced by ME (10 µM, 93±13.6 pA) prior to and the application of CTAP (1 µM, 8.9±3.9 pA, p=0.0013 paired T-Test, 6 cells, 3 animals). Download Figure 1-1, TIF file. [file eneuro-12-ENEURO.0249-24.2025-s001.tif]

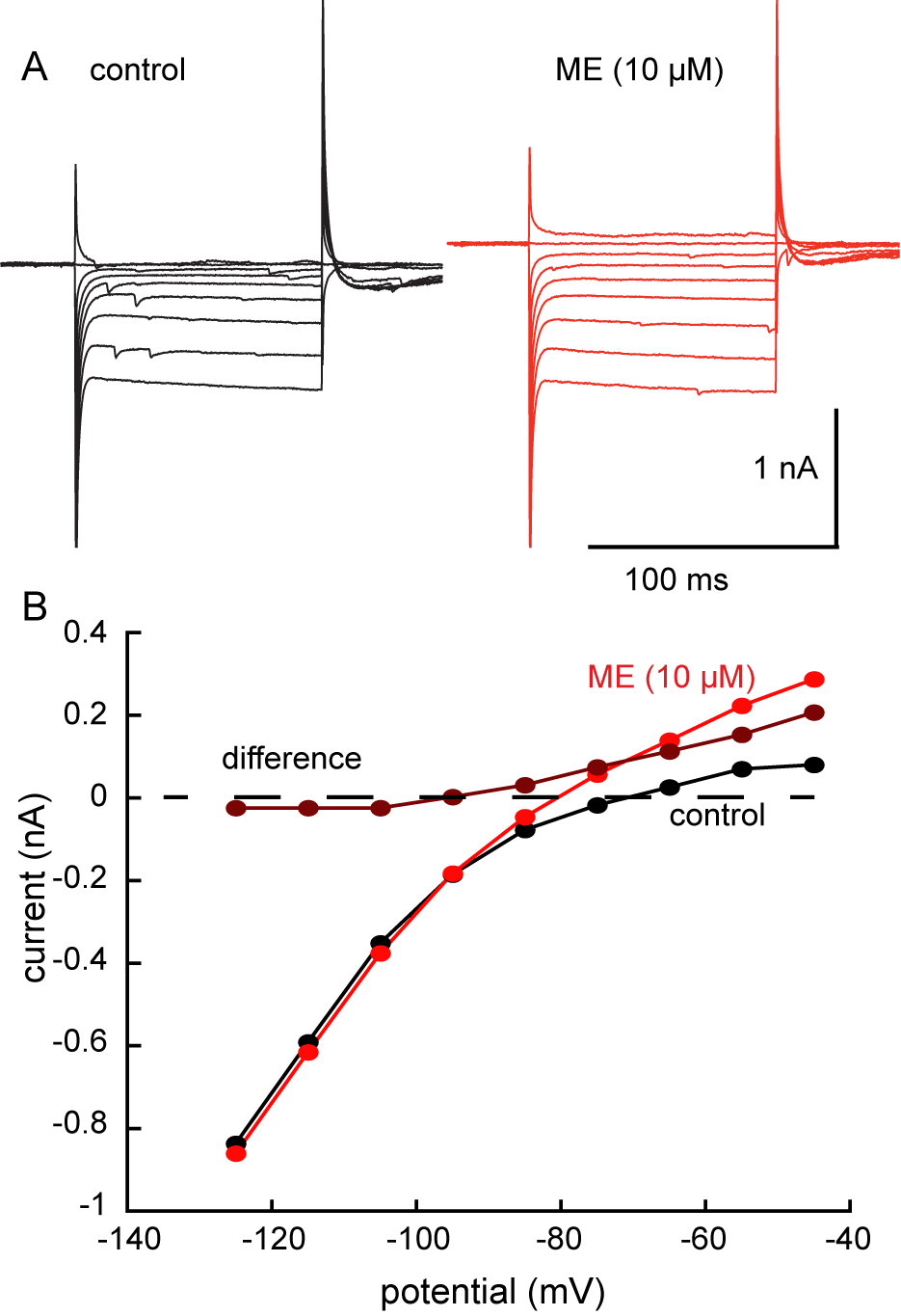

Supplement: Figure 2-1 — The outwardly rectifying potassium conductance measured with a voltage step protocol. A) The currents induced by voltage steps in the absence and presence of ME (10 µM). Voltage steps were made from -45 mV to -125 mV in 10 mV increments. B) The current voltage plots of the experiment illustrated in A. the ME current was determined by subtracting the control currents from those in the presence of ME. Download Figure 2-1, TIF file. [file eneuro-12-ENEURO.0249-24.2025-s002.tif]

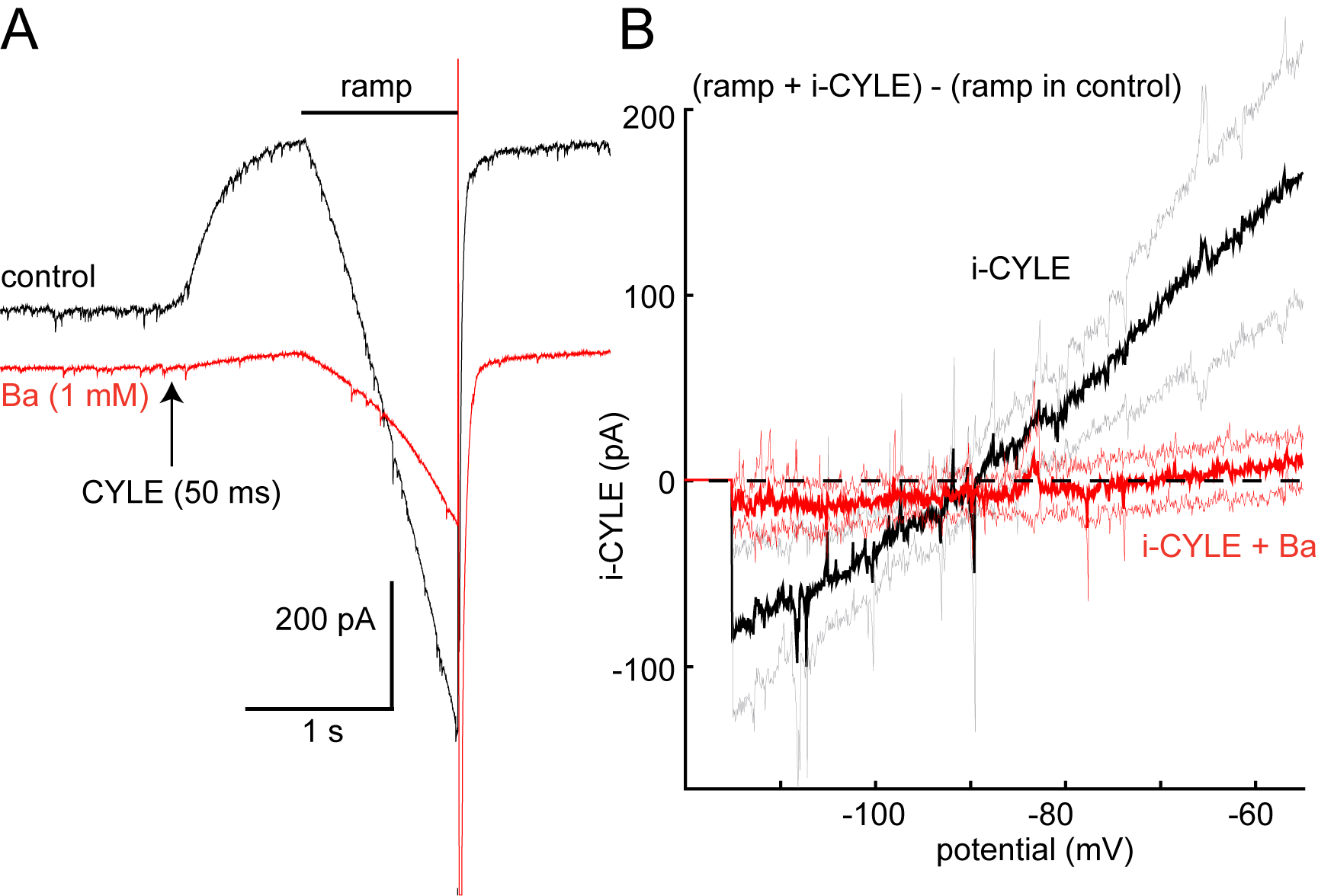

Supplement: Figure 2-2 — Barium blocks the opioid current induced by photoactivation of CYLE. A) The current measured during a ramp potential from -55 mV to -125 mV in the absence and presence of BaCl (1 mM). Arrow indicated the point of photoactivation of CYLE (50 ms). The addition of BaCl induced an inward current and blocked the outward current induced by CYLE. B) Summary of experiments with BaCl. The conductance induced by CYLE was blocked in the presence of BaCl (red). The dark lines indicate the mean current and the faint lines are the 95% CL (8 cells, 7 animals). Download Figure 2-2, TIF file. [file eneuro-12-ENEURO.0249-24.2025-s003.tif]

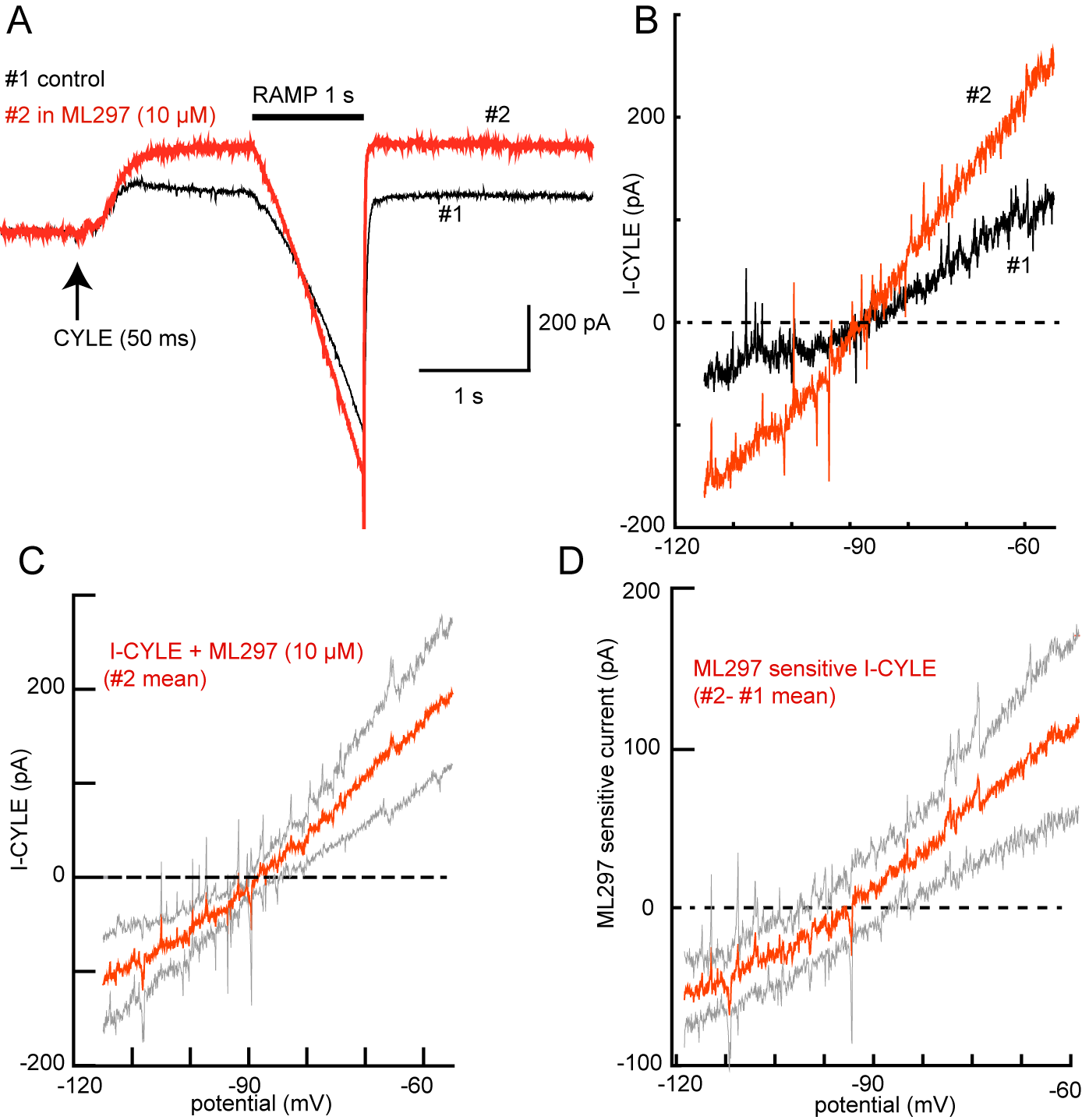

Supplement: Figure 2-3 — The potassium conductance is augmented in the presence of ML297 (10 µM). A) Example experiment illustrating the increase in CYLE induced current in the presence of ML297. B) The current voltage plot of the CYLE current obtained by subtracting the current illustrated in A) #1 from the current #2. The CYLE induced current was increased at all potentials. C) A summary of the current induced by CYLE in the presence of ML297 (from B #2). D) A summary of the ML297 sensitive current. The voltage dependence of the ML297 sensitive current (Part B #2-#1) is the same as that of the whole-cell current illustrated in part C. ML297 increased the conductance at all voltages though the current still rectified outwardly. (11 cells, 9 animals). Download Figure 2-3, TIF file. [file eneuro-12-ENEURO.0249-24.2025-s004.tif]

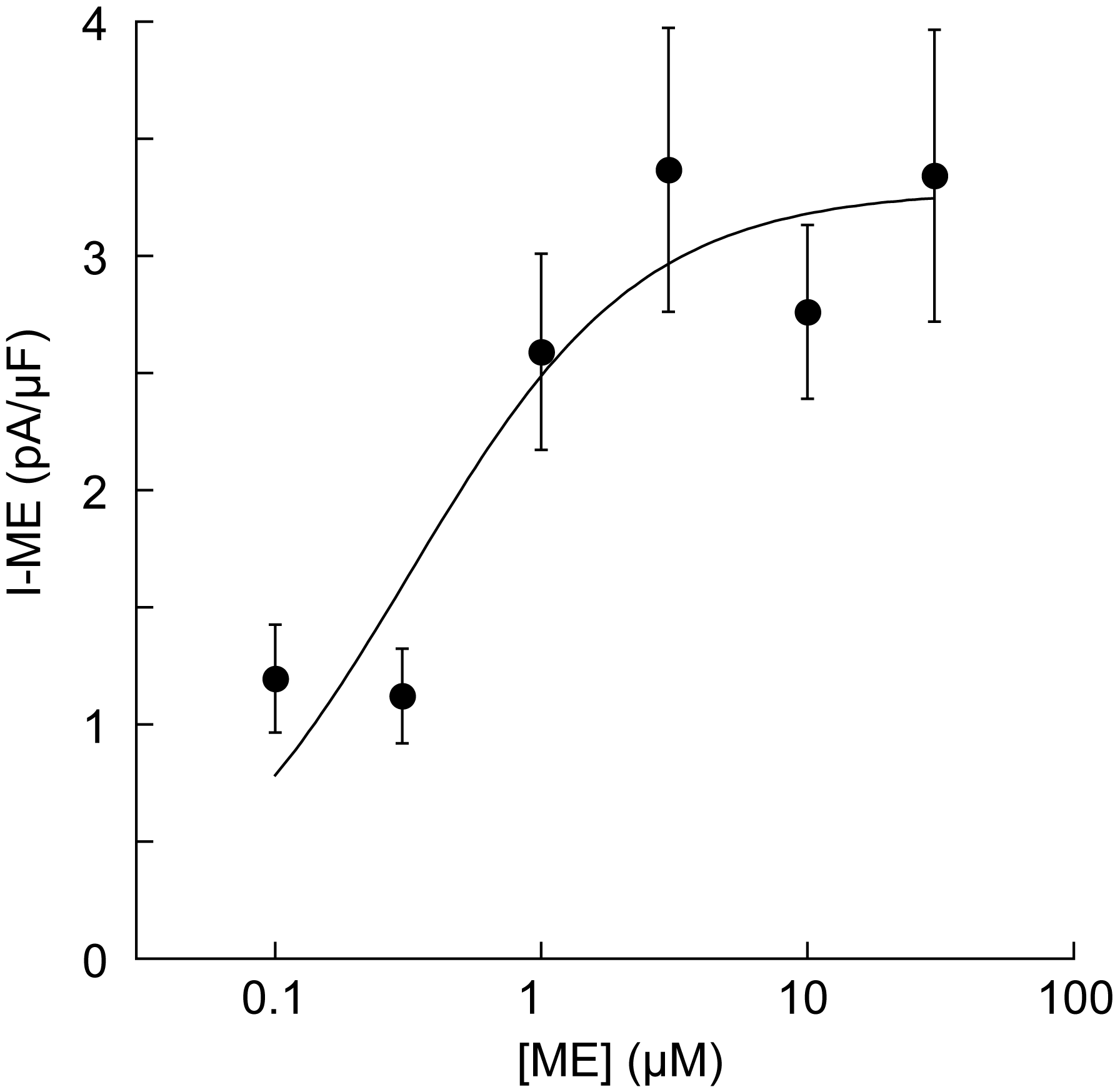

Supplement: Figure 4-1 — A concentration response curve to ME. The current amplitude was normalized to the capacitance of each neuron (current density, pA/µF). The plot indicates that ME applied at concentrations above 3 µM induce a maximum current. Download Figure 4-1, TIF file. [file eneuro-12-ENEURO.0249-24.2025-s005.tif]
